# Determination of the P-T conditions and depths of magma storage zones for two distinct quaternary lava flows in the Southeast of Mount Ağrı (Eastern Anatolia, Turkey)

**DOI:** 10.1371/journal.pone.0337393

**Published:** 2025-12-31

**Authors:** Gullu Deniz Dogan-Kulahci

**Affiliations:** Hacettepe University, Geological Engineering Department, Beytepe-Ankara, Turkey; National Autonomous University of Mexico Institute of Geophysics: Universidad Nacional Autonoma de Mexico Instituto de Geofisica, MEXICO

## Abstract

This study aims to determine the pressure (P)-temperature (T) conditions and depths of magma storage zones for two distinct lava flows located to the southeast of Mount Ağrı (5137 m), the highest stratovolcano in Turkey. The mineralogical–petrographic observations, whole-rock-trace and mineral chemistry, and geochronological (⁴⁰Ar/³⁹Ar dating) features of these lava flows were examined to provide the petrologic and temporal evolution of volcanic activity in the region. The flow referred to as Phase 1, with SiO₂ content ranging from 49.2% to 51.2%, was classified as trachybasalt, while the flow identified as Phase 2, with SiO₂ content between 62.42% and 63.4%, was classified as andesite. The whole-rock ^40^Ar/^39^Ar ages of samples from Phase 1 and Phase 2 were determined to be 57.70 ± 21.44 ka and 19.09 ± 5.59 ka, respectively. The older and more mafic Phase 1 flow displays a mineral assemblage of plagioclase + olivine ± oxide macrocrysts, microphenocrysts, and microlites. Conversely, the younger and more differentiated Phase 2 flow comprises a mineral assemblage of plagioclase + orthopyroxene ± clinopyroxene ± oxide macrocrysts, microphenocrysts, and microlites. Phase 1 is phaneritic with a scoriaceous texture and contains labradorite–composition plagioclase and Fo-rich olivine, whereas Phase 2 is aphanitic with a glassy texture, characterized by andesine–labradorite plagioclase, enstatite, and augite. EPMA analysis results and P-T calculations derived from equilibrated macrocrysts and microphenocrysts show that, assuming an average crustal density of 2.70 g/cm³, Phase 1 was emplaced at a depth of 38 km, with crystallization temperatures of 1186 °C from plagioclase data and approximately 1200 °C from olivine. In Phase 2, temperatures derived from plagioclase, clinopyroxene, and orthopyroxene are ~ 1128; 1147 and 1061 °C, with corresponding pressures of 8, 6, and 5 kbar, yielding calculated depths of 30 km, 23 km, and 19 km, respectively. These findings demonstrate that Phase 1 exhibits higher crystallization temperature, pressure, and depth values than Phase 2. Overall, the results reveal two distinct magma storage zones southeast of Mount Ağrı, active at different times during the Quaternary, which produced both mafic and intermediate volcanic products, with one located at a shallower and the other at a deeper level.

## 1. Introduction and geological setting

**Fig 1:**
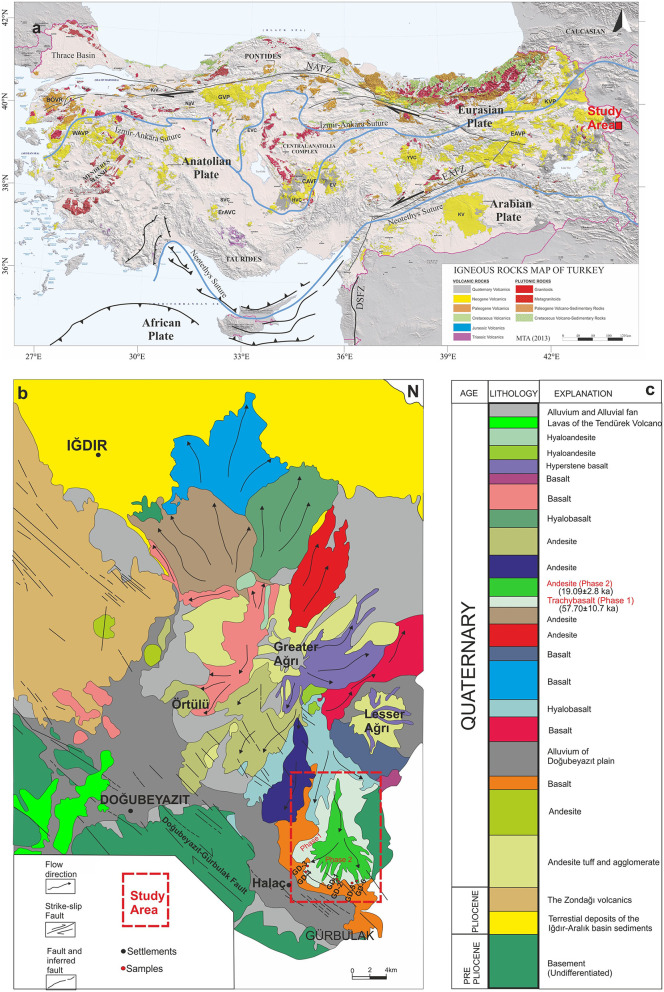
a) Location map of the study (Magmatic Rocks map of Turkey scale: 1/1.250.000 published by Mineral Research and Exploration of Turkey [8] and modified by [9]. EAHP: Eastern Anatolian High Plateau; NAFZ: North Anatolian Fault Zone; EAFZ: East Anatolian Fault Zone; DSFZ: Dead Sea Fault Zone; KVP: Kars Volcanic Plateau; EAVP: Eastern Anatolian Volcanic Province; PVP: Pontides Volcanic Province; CAVP: Central Anatolia Volcanic Province; GVP: Galatian Volcanic Province; KAVP: Kırka-Afyon Volcanic Province; WAVP: Western Anatolian Volcanic Province; YVC: Yamadağ Volcanic Complex; HVC: Hasandağ Volcanic Complex; EVC: Elmadağ Volcanic Complex; KVC: Konya Volcanic Complex; ErAVC: Erenlerdağ-Alacadağ Volcanic Complex. EV: Erciyes Stratovolcano; PV: Polatlı Volcanic Suite; SV: Sulutaş Volcanic Suite; NAV: Nallıhan Volcanic Suite; KıV: Kızılderbent Volcanic Suite; BOVR: Biga Orogenic Volcanic Rocks. Reprinted from [9] under a CC BY license, with permission from [Taylor & Francis] original copyright [2024]. b) Geological map of Mount Ağrı (modified from [3]. Reprinted from [3], under a CC BY license, with permission from [Elsevier] original copyright [1998].

**Fig 2:**
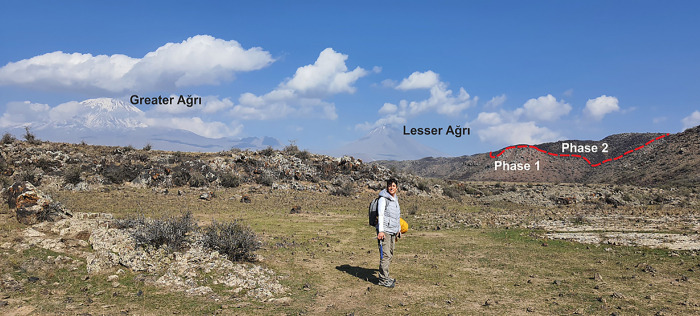
Outcrop-scale view of Greater and Lesser Ağrı, from Halaç.

Mount Ağrı (Mount Ararat) is an ice-capped, polygenic stratovolcano located in eastern Turkey near the borders of Iran and Armenia (Fig 1a). The edifice covers ~1100 km² with a dense rock equivalent (DRE) volume of ~1150 km³ and is characterized by two main volcanic cones, Greater Ağrı (5,137 m) and Lesser Ağrı (3,896 m), separated by a north–south-trending fault [1–5] (Fig 1b and Fig 2). A detailed overview of the geological formations in and around Mount Ağrı (Fig 1c). The volcano lies within a sinistral pull-apart basin controlled by strike-slip faults, particularly the Doğubeyazıt–Gürbulak and Iğdır Faults [2,6], which favored volcanism and influenced the current morphology of the region. The ongoing convergence between the Arabian and Eurasian Plates contributes to both volcanic and seismic hazards in the region [3,6,7].

Volcanic history of Mount Ağrı is complex, represented by 14 geologic units spanning from basalt to rhyolite but are commonly intermediate in composition (andesites and dacites) [10,11]. The more differentiated rocks are primarily located in the central part of Mount Ağrı, whereas the basaltic rocks are distributed along the flanks of the volcano [12]. The volcanism near Mount Ağrı initially exhibited a calc-alkaline character, and later evolved towards alkaline affinity over time [3,13–17]. According to [3], the volcanic evolution of Mount Ağrı can be divided into four primary phases (Fig 1b). (1) The initial stage of its volcanic history began with a Plinian eruption, followed by basaltic lava flows. Whole-rock K-Ar dating of basalt samples yielded an age of 1.51 ± 0.19 Ma [17], with their locations identified only from the “Van” map of [18] since coordinates were not available. The basaltic lava flows are considered to have originated from approximately NW-SE trending faults and fissures preceding the formation of the central volcano. (2) In the subsequent phases, the activity was primarily basaltic (K-Ar whole-rock ages 0.68 ± 0.24 Ma [19]), while the main cones developed through successive andesitic and dacitic eruptions (K-Ar whole-rock ages 0.5 Ma [17]). In addition, there are two more andesite ages from the northern part of the Greater Ağrı (K-Ar whole-rock ages 0.49–0.45 Ma ± 0.022 [20]. (3) At this stage, the crater of Greater Mount Ağrı began to rise above the pre-existing volcanic edifice, with lava erupting through fractures. During this time, the crater of Lesser Mount Ağrı also reached its full development. (4) In the final stage a major north-south fault separates the Greater Ağrı from the Lesser Ağrı, creating a gap that facilitates magma extrusion. Flank eruptions (K-Ar whole-rock ages between 0.30 ± 0.33 Ma for basalt and <0.049 Ma for andesite Ma [17,19–21] along this fault system resulted in the formation of numerous parasitic cones, domes, and mudflows. In addition to earlier studies, [22] reported basaltic lavas from the Maku area (NW Iran, ~ 50 km away), with ⁴⁰Ar/³⁹Ar ages of 0.81 ± 0.10 Ma, 0.40 ± 0.05 Ma, and 0.48 ± 0.03 Ma. Similar to the present work, their samples also contained low radiogenic argon. The youngest known K-Ar age is obtained for an andesitic rock (<0.02 Ma) reported by [21], however, information on the location of this sample is not available as well.

A recent study [12] examined one of these flows to the southeast of Lesser Ağrı, north of the Halaç settlement (Fig 1b) through numerical modeling. The numerical models developed and analyzed by [12] were implemented using finite element software (COMSOL; see also [23,24]. The models are constructed as two-dimensional representations based on the geological framework of the Mount Ağrı volcano, derived from field observations, seismic wave profiles, and InSAR data [25]. Magma chambers and reservoirs are represented as cavities with applied boundary loads to simulate magmatic overpressure conditions [26,27]. Their main objective is to find the plumbing system and distribution of melt storage zones. Through this model, they determined the covered area and the volume of the lava deposits and estimated the depths of potential magma storage systems feeding Mount Ağrı. Based on that study, in the southwest part of Lesser Ağrı there are two generations of basaltic lava flows, the earlier eruption covered an area of about 96 km² and had a volume of approximately 3.2 km³, while the later eruption was smaller, with an area of 25 km² and a volume of 0.6 km³. The earlier is a large, deeper reservoir located at a depth of 20 km, while the latter is a shallower magma chamber situated at 8 km depth. All the lava flows are considered to have the same geochemical characteristics, which [3] initially classified as “andesitic flows”, while most recently [12] grouped as “recent basaltic flows 1 and 2”. [12] noted that the lack of petrogenetic and geochemical data for the two lava flows examined in their study makes it challenging to understand the volcanic evolution in the south of the Mount Ağrı. Based on the recommendations for future research provided in their work, the present study aims to address these gaps through a comprehensive approach that includes mineralogical and petrographic investigations, mineral chemistry analyses using electron probe microanalysis (EPMA), whole-rock and trace element geochemistry, and ^40^Ar/^39^Ar radiometric dating of samples collected from both lava flows. The primary objective of this study is to identify the macro and micro scale differences between Phase 1 and Phase 2 lava flows, determine the pressure-temperature (P-T) conditions and depths of the associated magma storage zones using EPMA data. These results are then compared with the numerical models of [12] and with previous studies on the volcanic evolution of the region. Additionally, the whole-rock ^40^Ar/^39^Ar dating method applied to these two distinct lava flows provides temporal constraints on the evolution of the volcanic activity in the region. Such studies are considered significant due to the limited number of research available on the chronological evolution of the volcanic activity in stratovolcanoes across Eastern Anatolia, including Mount Ağrı and its neighboring volcanoes. Considering the tectonic activity within the region, the associated risk of lahar flow [28], the proximity to the Karlıova triple junction [12], and the high population density in the adjacent urban areas [29], the critical importance of the region is further emphasized.

## 2. Analytical methods

**Fig 3:**
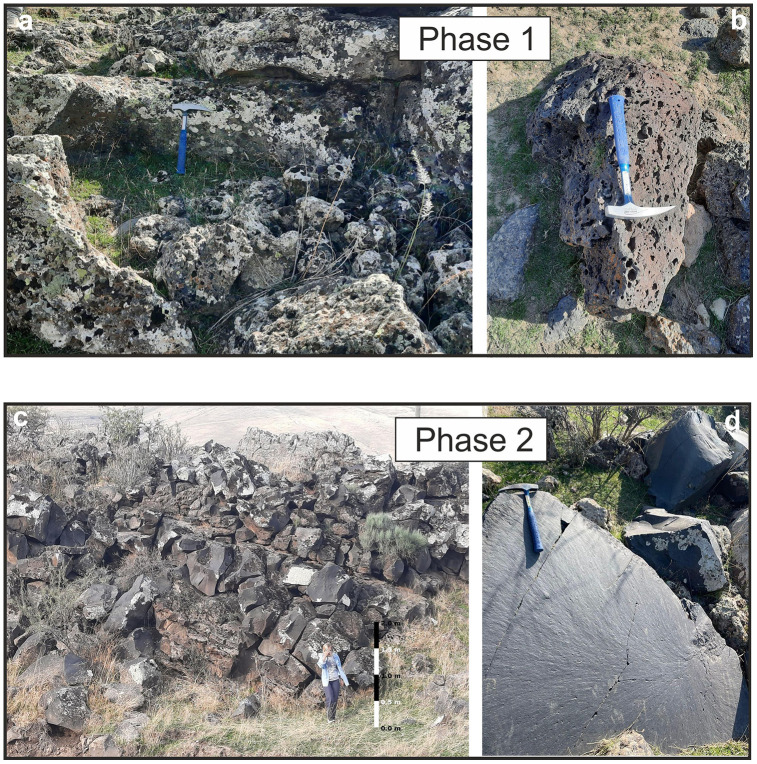
(a) Outcrop-scale view of Phase 1. (b) Close-up view of Phase 1, characterized by a gray color and scoriaceous texture. (c) Outcrop-scale view of Phase 2 with a 1.70-meter-tall person (right bottom). (d) Close-up view of Phase 2, which is black and exhibits a smooth, glass-like surface.

Electron Microprobe Analysis (EPMA) was conducted at the Earth Sciences Application and Research Center (YEBIM) laboratory, at Ankara University. Polished thin sections prepared for EPMA were carbon-coated and then examined using a JEOL JXA-8230 (WDS) electron microscope. Microprobe analyses were performed on plagioclase, olivine, orthopyroxene and clinopyroxene macrocrysts, microphenocrysts, and microlites. The analyses were carried out under conditions of 20 kV accelerating voltage, 20 nA beam current, and 5 µm beam size have been used for both basalt and andesite samples. Scanning Electron Microscope (SEM) micrographs were obtained at YEBIM. Optical microscope studies were conducted at the Department of Geological Engineering, Hacettepe University. Whole-rock and Trace element analyses were carried out at ALS Scandinavia AB labs (Sweden). A lithium borate fusion prior to acid dissolution and ICP-MS analysis provides the quantitative analytical approach for a broad suite of trace elements. All whole-rock results were obtained from analyses conducted using ICP-AES with lithium borate fusion [30]. Cation calculations from the mineral chemistry data were performed using the “Excel-based Mineral Classification and Geothermobarometry Program for Magmatic Rocks” developed by [31]. The Ar-Ar age analyses of the samples were performed at the New Mexico Geochronology Research Laboratory in the USA. Samples were crushed and sieved to a size appropriate for the preparation of high-alunite separates. The samples were irradiated at the Oregon State, TRIGA reactor Oregon – USA. The Fish Canyon tuff standard with an age of 28.201 ± 0.023 Ma [32] was used as a neutron flux monitor. The J-value for each sample was determined by a combination of eight to ten single-grain laser fusion analyses located in 6 radial positions around the irradiation disc. The J-value is a flux parameter derived from the irradiation of a standard mineral of known age, which calibrates the neutron flux experienced by the unknown samples [33]. Correction factors for interfering neutron reactions were determined using co-irradiated CaF2 and K-glass. The total decay constant of 5.463e-10/a [34] and the ^40^Ar/^36^Ar atmospheric ratio at 295.5 ± 0.5 [35] were used for age calculations. The samples were analyzed by the incremental step-heating method using a defocused diode laser to heat the samples, and argon isotopes were measured using a ThermoFisher Scientific Helix MC multi-collector mass spectrometer. For some samples, two aliquots have been analyzed. Data reduction and age assignment are based on Pychron Software [36], and uncertainties are reported at the 2s confidence level. The individual pictured in Fig 3 has provided written informed consent (as outlined in PLOS consent form) to publish their image.

### 2.1. Inclusivity in global research

Additional information regarding the ethical, cultural, and scientific considerations specific to inclusivity in global research is included in the Supporting Information.

## 3. Mineralogy and petrography of the two distinct lava flows

Preliminary studies revealed that two lava flows with distinct colors (Fig 1b) are located in the southwestern part of Lesser Ağrı, near the village Halaç (Fig 2). Six fresh hand specimens were collected in order to obtain petrological data from these lava flows. The samples are classified into Phase 1 and Phase 2 based on mineralogical-petrographic examinations and geochemical analyses. Phase 1 is characterized by a gray color with a scoriaceous texture, while Phase 2 is black with a smooth, glass-like surface (Fig 3).

### 3.1. Phase 1

**Fig 4:**
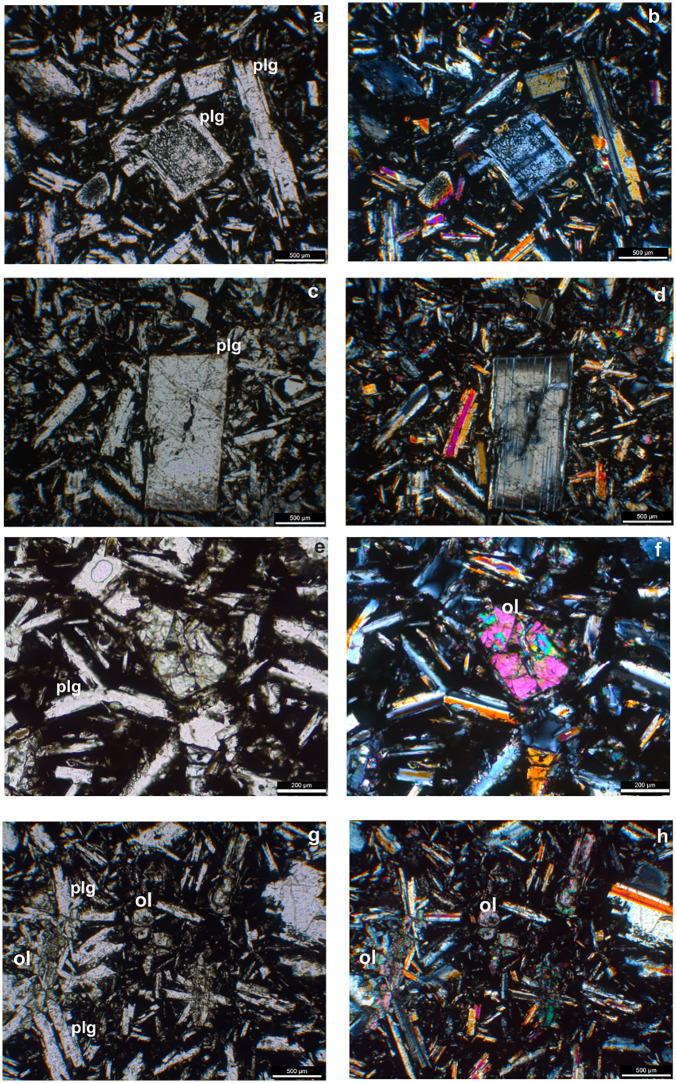
Thin section microphotographs of Phase 1 flow. **(a, b)** Plagioclase (plg) macrocrysts with sieved cores and homogeneous rims **(c, d)**. Plagioclase macrocrysts with zoned pattern **(e, f)**. Fragmented olivine (ol) microphenocryst **(g, h)**. Hypocrystalline porphyritic textures of the rock, and glomerocryst of plagioclase microphenocrystals (a, c, e and g are plane-polarized light; b, d, f and hare and cross-polarized crossed light).

The older Phase 1 flow is characterized by a mineral assemblage comprising plagioclase and olivine ± oxides. Plagioclase and olivine predominantly occur as macrocrysts (≤ 500 µm – < 10 mm length; ≥ 30 µm width [37], microphenocrysts (≤ 100 µm – < 500 µm length; ≥ 30 µm width), whereas oxides are mainly present as microlite (≥1 µm width) (Fig 4). Plagioclase macrocrysts exhibit sieved cores and homogeneous rims (Fig 4a, 4b), alongside oscillatory zoning pattern (Fig 4c, 4d). All olivine crystals are fragmented (Fig 4e, 4f). Phase 1 displays a hypocrystalline porphyritic to glomeroporphyritic texture consisting of plagioclase microphenocrysts (Fig 4g, 4h), and medium-grained microlites are observed within the in groundmass.

### 3.2. Phase 2

**Fig 5:**
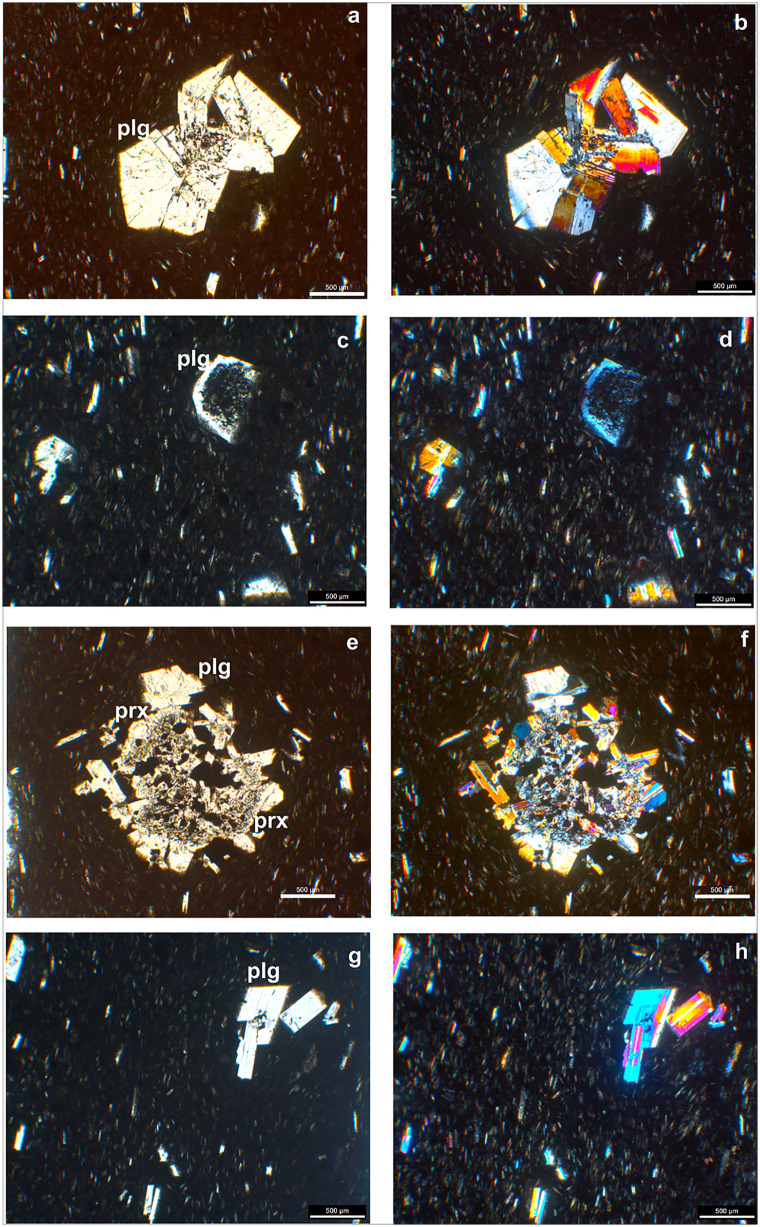
Thin section microphotographs of Phase 2 flow. **(a, b)** Glomerocryst of plagioclase (plg) macrocrysts and microlites. **(c, d)** Plagioclase macrocrysts with sieved core and homogeneous rim. **(e, f)** Glomerocryst Plagioclase and orthopyroxene bearing glomerocryst. **(g, h)** Black matrix composed of fine-grained plagioclase microlites. (a, c, e and g are plane-polarized light; b, d, f and hare and cross-polarized crossed light).

Phase 2 flow comprises a mineral assemblage of plagioclase + orthopyroxene ± clinopyroxene ± oxides. Plagioclase and orthopyroxene predominantly occur as macrocrysts and microphenocrysts, whereas clinopyroxene and oxides are mainly observed as microlites (Fig 5). Plagioclase macrocrysts and microphenocrysts sometimes occur as crystal aggregates (Fig 5a, 5b), with the microphenocrysts are commonly display sieved cores surrounded by compositionally homogeneous rims which in back-scattered electron (BSE) images exhibit oscillatory zoning (Fig 5c, 5d). Similar to Phase 1, Phase 2 flows exhibit hypocrystalline porphyritic texture (Fig 5e, 5f), with a glassy groundmass composed of fine-grained microlites (Fig 5g, 5h).

## 4. Geochemistry

Six rock samples from the southwestern flank of Mount Ağrı were analyzed for major elements. Two samples, one from each phase were analyzed for trace elements. Phase 1 and Phase 2 samples are fresh and characterized low loss on ignition (LOI) values (<1 wt%) (Table 1). Phase 1, characterized by SiO₂ contents ranging from 49.2–51.2 wt.% and total alkali (Na₂O + K₂O) values between 5–6 wt.%.

**Table 1:** Whole rock and Trace element compositions of Phase 1 and Phase 2 samples.

	Sample Name	SiO_2_	TiO_2_	Al_2_O_3_	Fe_2_O_3_	MnO	MgO	CaO	Na_2_O	K_2_O	P_2_O_5_	LOI	TOTAL
**Phase 1**	**GD-3**	51,20	1,68	17,70	10,05	0,14	5,59	7,90	4,33	0,64	0,29	0,10	99,62
	**GD-4**	49,21	1,86	19,69	10,39	0,16	3,64	9,26	4,43	0,61	0,31	0,10	99,66
	**GD-5**	50,06	1,76	20,00	9,65	0,15	3,14	9,20	4,58	0,63	0,28	0,30	99,75
**Phase 2**	**GD-6**	62,42	0,76	17,67	5,08	0,10	0,86	5,11	4,78	1,96	0,29	0,70	99,73
	**GD-1**	63,40	0,74	16,80	5,88	0,09	1,50	4,54	4,69	1,86	0,30	0,10	99,90
	**GD-2**	62,37	0,78	17,64	5,37	0,11	1,11	5,22	4,82	1,97	0,29	0,10	99,78
		**Ba**	**Ce**	**Cr**	**Cs**	**Dy**	**Er**	**Eu**	**Ga**	**Gd**	**Hf**	**Ho**	**La**
**Phase 1**	**GD-3**	124,50	24,60	49	0,20	4,16	2,36	1,34	17,60	4,18	3,64	0,91	10,60
**Phase 2**	**GD-1**	545,00	47,20	5	1,22	3,36	2,12	1,14	19,60	3,83	4,85	0,74	26,10
		**Lu**	**Nb**	**Nd**	**Pr**	**Rb**	**Sc**	**Sm**	**Sn**	**Sr**	**Ta**	**Tb**	**Th**
**Phase 1**	**GD-3**	0,32	5,22	15,00	3,36	7,90	21,30	3,95	1,50	411	0,50	0,68	1,34
**Phase 2**	**GD-1**	0,28	12,95	20,60	5,11	43,20	10,70	4,53	1,20	417	0,90	0,56	5,95
		**Ti**	**Tm**	**U**	**V**	**W**	**Y**	**Yb**	**Zr**				
**Phase 1**	**GD-3**	0,96	0,35	0,42	165	<0.5	23,20	2,23	158				
**Phase 2**	**GD-1**	0,51	0,28	2,12	72	1,4	20,80	1,97	199				

**Fig 6:**
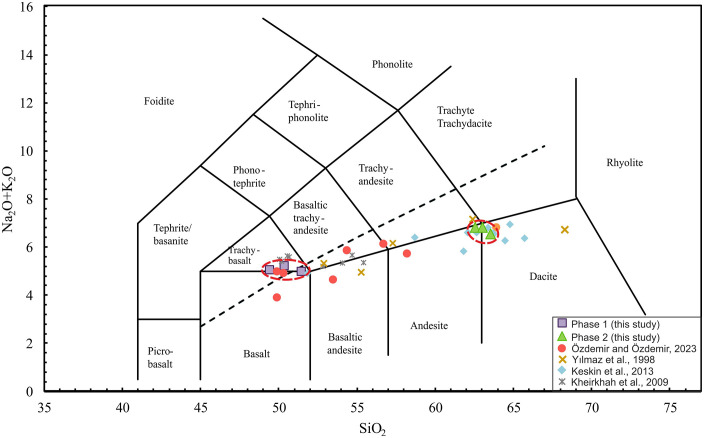
Total Alkaline (Na_2_O+K_2_O wt%) vs. silica (SiO_2_ wt%) TAS diagram [41]. (dashed line, according to [42]. The comparison data are from (Cumaçay volcanics 50 km away from Ağrı- [40]; Mount Ağrı- [3], Mount Ağrı- [39] and Mount Ağrı, [38].

Phase 1 samples display a transitional character, plotting within the trachy-basalt to basaltic trachyandesite fields with moderate alkalinity (Fig 6). They are characterized by elevated MgO (3–5.6 wt.%) and Fe₂O₃ (9–10 wt.%) contents, consistent with the crystallization of mafic minerals such as olivine. In contrast, Phase 2 samples have higher SiO₂ contents (62.4–63.4 wt.%), and total alkalis of ~6–7 wt.%, plotting as andesite and exhibits a sub-alkaline affinity (Fig 6). They are marked by lower MgO (0.8–1.5 wt.%) and Fe₂O₃ (5–6 wt.%) contents, indicative of differentiation. For comparison, the samples obtained in this study were compared with geochemical data from previous investigations on Mount Ağrı and its vicinity [3,38–40].

**Fig 7:**
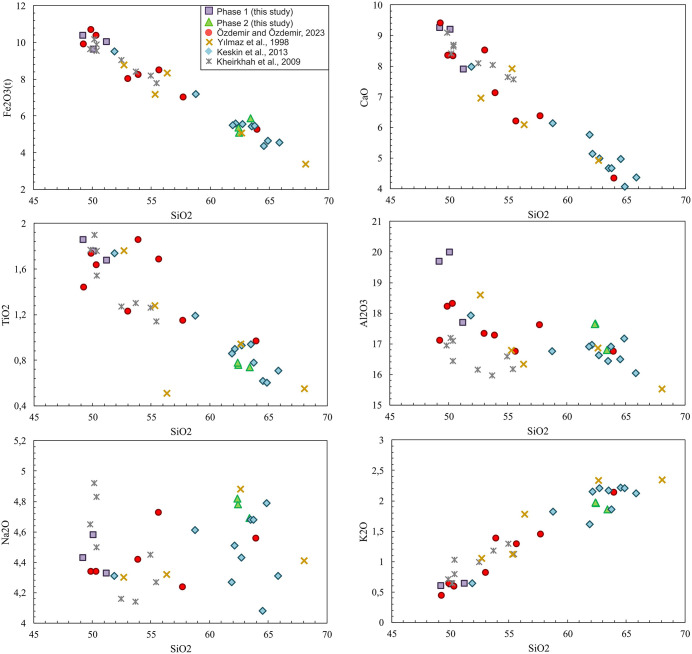
Selected major variations diagrams of Phase 1 (this study) and Phase 2 (this study). The comparison data are the same as those presented in Fig 6.

On Harker diagrams (Fig 7), Phase 1 has high CaO, FeO, TiO_2_, Al_2_O_3_ and low K₂O and Na_2_O concentrations. Phase 2 elevated K₂O and Na_2_O contents imply enrichment in plagioclase and alkali feldspar. The consistent correlations among the selected major element compositions indicate that the lava flows in the southeastern part of Mount Ağrı were influenced by magmatic differentiation processes.

**Fig 8:**
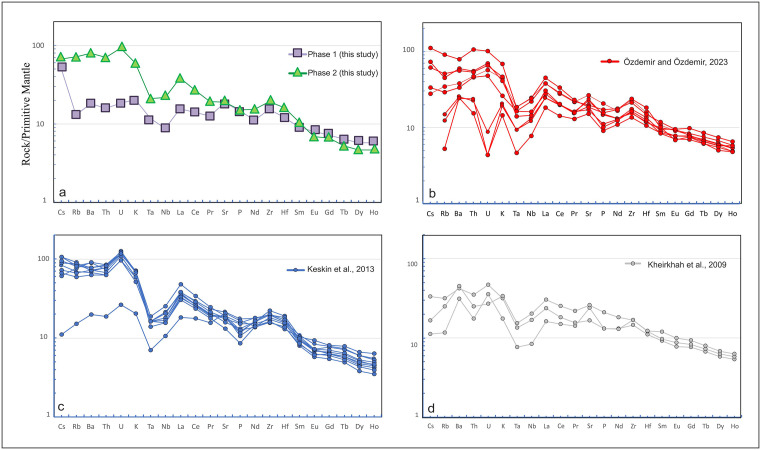
Primitive mantle (PM)-normalized [43] trace element plots for Phase 1 and Phase 2, comparing samples from Mount Ağrı [38,39] and Cumaçay volcanics [40].

The primitive mantle-normalized multi-element diagram (Fig 8a) reveals distinct geochemical patterns between Phase 1 and Phase 2 samples. Phase 2 displays elevated concentrations particularly LILEs (e.g., Cs, Rb, Ba, Th, and U), suggesting an enrichment in incompatible components. The negative Nb and Ta anomalies observed in both phases. Light rare earth elements (LREEs; La to Sm) also show a higher enrichment in Phase 2 compared to Phase 1, while both phases exhibit relatively flat patterns in the heavy rare earth elements (HREEs; Gd -Ho). When compared with published data, Cumaçay volcanics of [40] show affinities with Phase 2, [39] with both phases, and [38] with Phase 1.

**Fig 9:**
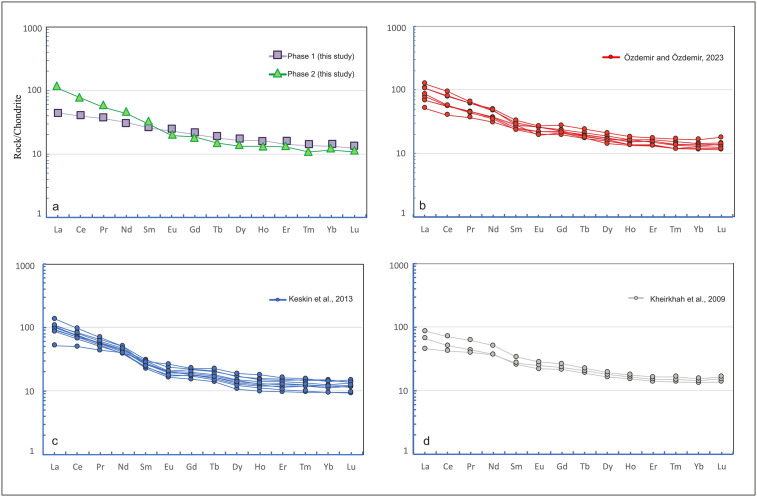
Chondrite-normalized [43] trace element plots for Phase 1 and Phase 2, comparing samples from Mount Ağrı [38,39] and Cumaçay volcanics [40].

According to the REE concentrations (Fig 9), Phase 2 is enriched in LREE (La–Sm) and a flat trend in HREE (Gd–Lu). The absence of a clear Eu anomaly indicates limited plagioclase fractionation. Phase 1 samples, although comparable to Phase 2 in terms of LREE enrichment, show lower abundances.

## 5. Ar-Ar dating

**Fig 10:**
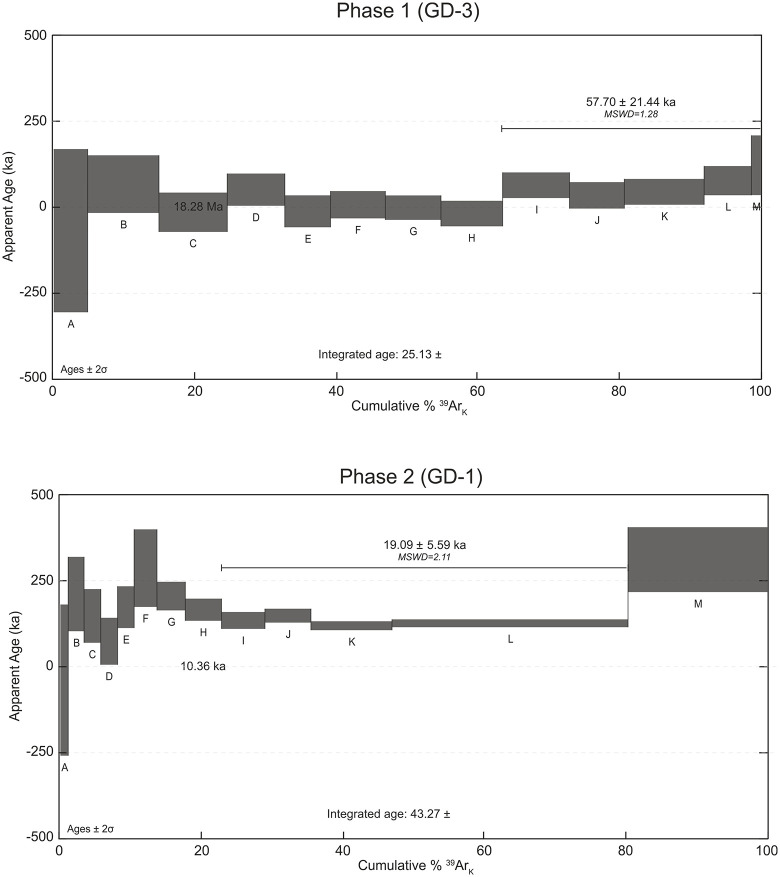
Age spectra for the sample GD-3 from Phase 1 and GD-1 from Phase 2. The plateau ^40^Ar-^39^Ar age show and the integrated ^40^Ar-^39^Ar age are also presented on the age spectra.

Geochemical studies revealed that the chemical compositions of Phase 1 and Phase 2 are distinct, emphasizing the importance of dating these lava flows. Accordingly, two representative fresh samples, GD-3 (Phase 1) and GD-1 (Phase 2), were selected for whole-rock ⁴⁰Ar/³⁹Ar isotopic dating. Sample GD-1 presents a well-defined plateau age, supported by at least three consecutive heating steps with overlapping ages within 2σ uncertainty and accounting for over 50% of the released ³⁹Ar (e.g., [44]). In contrast, sample GD-3 displays an overall flat spectrum with a very low radiogenic yield (a very small proportion of ⁴⁰Ar* relative to total argon). The flat part at the highest temperature steps yields an age of 57.70 ± 21.44 ka (Fig 10). These ages indicate that the lava flows defined as Phase 1 and Phase 2 are Quaternary aged samples. Both samples exhibited low radiogenic yields, with the basaltic sample (GD-3) yielding less than 2% radiogenic isotopes, a characteristic also observed in the Maku basalts located 50 km from the present study area. The low radiogenic yield invalidates the inverse isochrone for both samples.

## 6. Mineral chemistry

**Fig 11:**
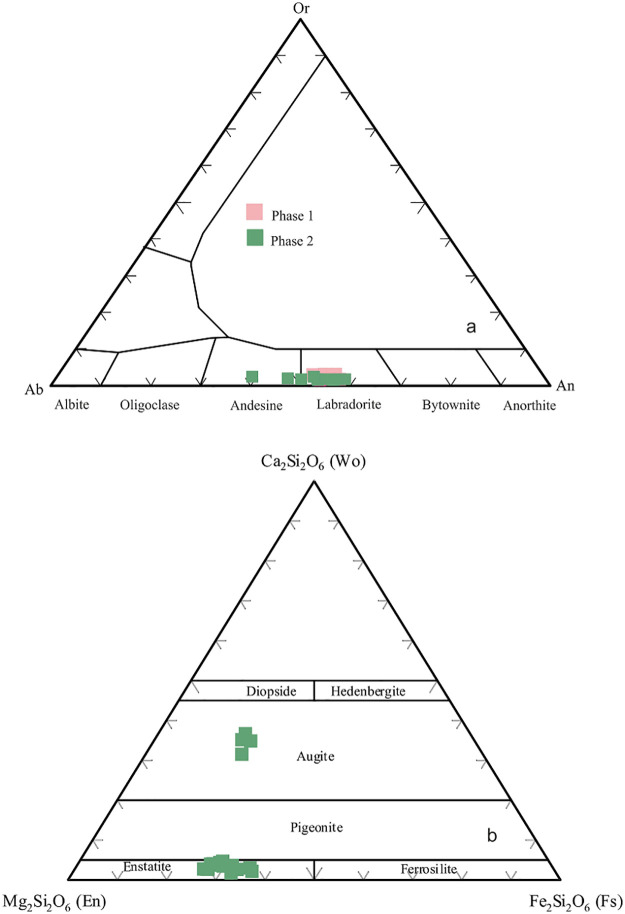
a) Compositions of feldspars from Phase 1 and Phase 2. b) Compositions of pyroxenes from Phase 1 and Phase 2.

**Fig 12:**
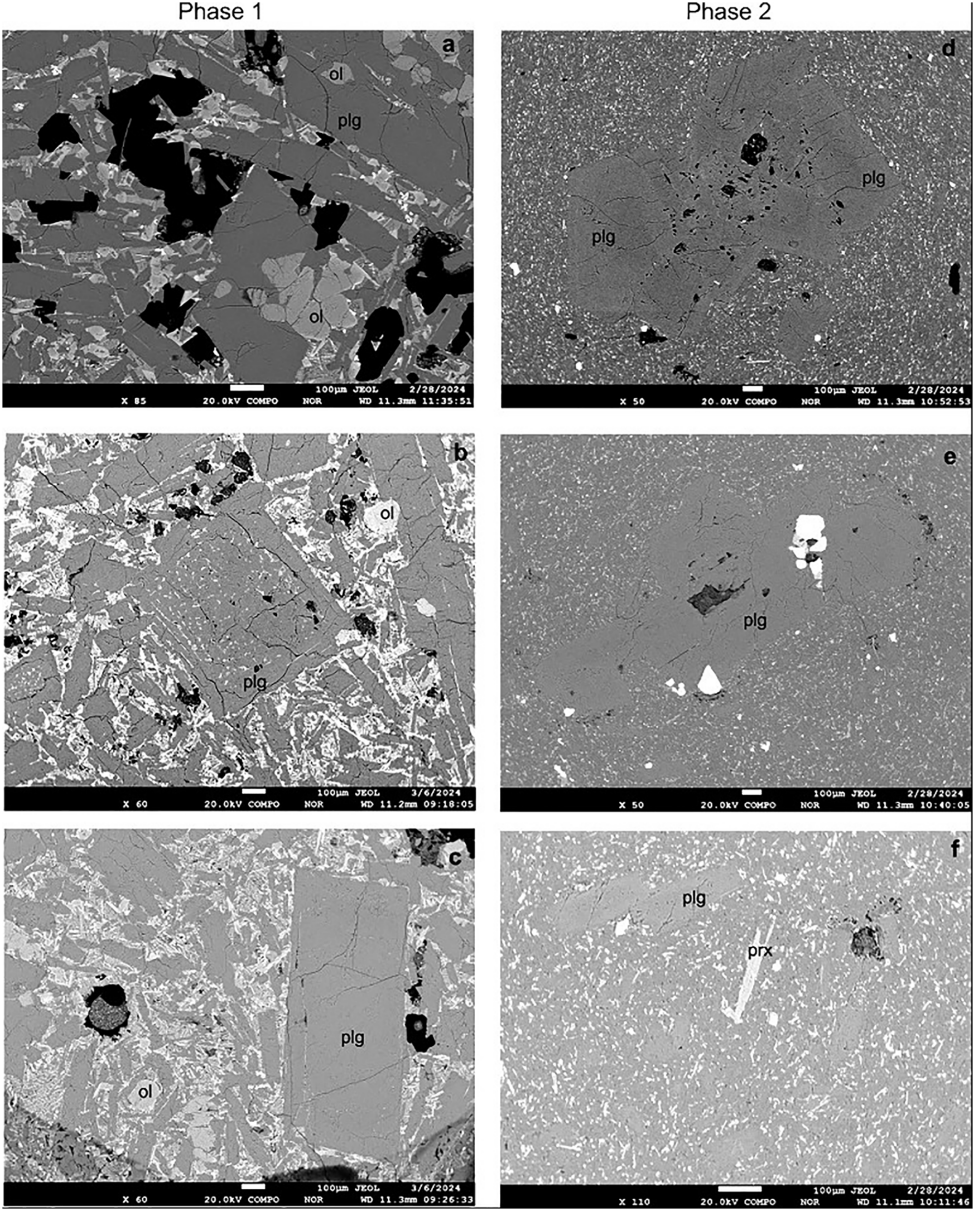
a, b, c). SEM images of the plagioclase, olivine macrocrysts, microcrysts, and microlites in the groundmass of Phase 1 samples. d, e, f) SEM images of the plagioclase, pyroxene phenocrysts, microcrysts and microlites in the groundmass of the Phase 2 samples.

In Phase 1, plagioclase macrocrysts and microphenocrysts with An_(52–58)_ exhibit a compositional range classified as labradorite (Fig 11a). SEM images (Fig 12) illustrate the groundmass textures of Phase 1 and Phase 2. While Phase 1 has medium-grained microlites in groundmass and the plagioclase and olivine crystals are larger in size, whereas Phase 2 has fine grained microlites (Fig 12). Plagioclase macrocrysts and microphenocrysts in Phase 2 exhibit compositions ranging from An₃₉ to An₅₈, indicating a transitional composition between labradorite and andesine (Table 3; Fig 11a). Although plagioclases predominantly exhibit normal zoning, some crystals display reverse zoning characterized by anorthite-rich rims (Table 2 and Table 3).

**Table 2:** Selected microprobe analyses of plagioclase on Phase 1 samples (c: core. r: rim) (Calculated based on 32 O).

Plagioclase	GD-3														GD-4			GD-5	
	A1-2c	A3-1c	A3-2c	A4-2c	A4-2r	A5-1c	A5-2c	A6-1c	A7-2c	A8-1c	A8-1r	A9-1c	A11-1c	A12-2c	A1-2c	A2-3c	A5-2c	A3-1c	A7-1c
**SiO**_**2**_	55,30	55,01	53,70	54,05	52,53	53,72	53,87	54,45	59,46	55,01	55,50	56,28	56,14	54,92	54,58	54,42	55,12	54,59	54,49
**TiO**_**2**_	0,10	0,11	0,09	0,13	0,09	0,01	0,14	0,04	0,07	0,11	0,06	0,08	0,14	0,16	0,09	0,06	0,11	0,05	0,06
**Cr**_**2**_ **O**_**3**_	0,00	0,00	0,00	0,00	0,00	0,00	0,00	0,00	0,00	0,00	0,00	0,00	0,00	0,00	0,00	0,00	0,00	0,00	0,00
**Al**_**2**_ **O**_**3**_	27,03	26,46	27,96	27,79	28,93	28,06	27,72	27,86	22,92	26,60	25,89	26,43	25,73	27,09	28,00	28,35	27,01	28,32	28,18
**FeO**	0,39	0,36	0,34	0,38	0,43	0,49	0,41	0,40	0,35	0,43	0,41	0,49	0,34	0,40	0,33	0,41	0,39	0,33	0,40
**MnO**	0,00	0,00	0,00	0,00	0,00	0,00	0,00	0,00	0,00	0,00	0,00	0,00	0,00	0,00	0,00	0,00	0,00	0,00	0,00
**MgO**	0,14	0,08	0,14	0,12	0,14	0,09	0,10	0,10	0,03	0,18	0,10	0,13	0,09	0,10	0,11	0,08	0,16	0,12	0,15
**CaO**	11,60	11,31	11,84	11,63	11,83	11,08	11,55	11,78	11,81	12,21	11,86	11,21	11,74	11,60	11,38	11,68	12,04	11,72	11,66
**K**_**2**_ **O**	0,11	0,10	0,11	0,11	0,12	0,14	0,10	0,11	0,12	0,10	0,09	0,13	0,09	0,10	0,09	0,09	0,11	0,09	0,11
**Na**_**2**_ **O**	5,27	5,23	5,32	5,22	4,99	5,73	5,33	4,93	5,14	5,59	5,16	5,16	4,76	4,93	5,40	4,68	4,98	4,70	4,90
**Total**	99,94	98,66	99,49	99,41	99,05	99,32	99,22	99,67	99,90	100,22	99,07	99,91	99,03	99,30	99,98	99,76	99,93	99,92	99,93
**Si** ^**4+**^	10,00	10,09	9,73	9,82	9,57	9,72	9,80	9,89	10,85	9,90	10,15	10,21	10,31	10,03	9,85	9,89	9,99	9,90	9,87
**Ti** ^**4+**^	0,01	0,01	0,01	0,02	0,01	0,00	0,02	0,01	0,01	0,01	0,01	0,01	0,02	0,02	0,01	0,01	0,01	0,01	0,01
**Cr** ^**3+**^	0,00	0,00	0,00	0,00	0,00	0,00	0,00	0,00	0,00	0,00	0,00	0,00	0,00	0,00	0,00	0,00	0,00	0,00	0,00
**Al** ^**3+**^	5,76	5,72	5,97	5,95	6,21	5,99	5,94	5,96	4,93	5,64	5,58	5,65	5,57	5,83	5,95	6,07	5,77	6,06	6,02
**Fe** ^**2+**^	0,06	0,05	0,05	0,06	0,06	0,07	0,06	0,06	0,05	0,06	0,06	0,07	0,05	0,06	0,05	0,06	0,06	0,05	0,06
**Mn** ^**2+**^	0,00	0,00	0,00	0,00	0,00	0,00	0,00	0,00	0,00	0,00	0,00	0,00	0,00	0,00	0,00	0,00	0,00	0,00	0,00
**Mg** ^**2+**^	0,04	0,02	0,04	0,03	0,04	0,02	0,03	0,03	0,01	0,05	0,03	0,04	0,03	0,03	0,03	0,02	0,04	0,03	0,04
**Ca** ^**2+**^	2,25	2,22	2,30	2,26	2,31	2,15	2,25	2,29	2,31	2,35	2,32	2,18	2,31	2,27	2,20	2,28	2,34	2,28	2,26
**Na** ^**+**^	1,85	1,86	1,87	1,84	1,76	2,01	1,88	1,74	1,82	1,95	1,83	1,81	1,69	1,74	1,89	1,65	1,75	1,65	1,72
**K** ^**+**^	0,03	0,02	0,02	0,03	0,03	0,03	0,02	0,03	0,03	0,02	0,02	0,03	0,02	0,02	0,02	0,02	0,03	0,02	0,02
**Ab**	44,82	45,30	44,61	44,53	43,01	47,95	45,24	42,83	43,74	45,09	43,84	45,11	42,10	43,20	46,00	41,80	42,55	41,86	42,92
**An**	54,56	54,14	54,81	54,85	56,34	51,27	54,20	56,55	55,57	54,39	55,64	54,17	57,38	56,22	53,52	57,69	56,81	57,62	56,47
**Or**	0,62	0,56	0,58	0,61	0,65	0,79	0,56	0,62	0,69	0,52	0,52	0,72	0,52	0,58	0,48	0,51	0,64	0,52	0,62
**Kd (Ab-An)** ^**plg-liq**^ **=(0.28 ± 0.11)**	***0,34***	***0,34***	***0,33***	***0,33***	0,31	***0,38***	***0,34***	***0,31***	***0,32***	***0,34***	0,32	***0,34***	***0,30***	***0,32***	0,47	***0,39***	0,41	***0,38***	0,40
**T(°C) Eq.24a (± 36)**	***1218***	***1217***	***1218***	***1196***		***1190***	***1180***	***1185***	***1183***	***1146***		***1146***	***1151***	***1149***		***1221***		***1199***	
**P (kbar) Eq. 25a (± 2,5)**	***10***	***10***	***10***	***9***		***10***	***9***	***8***	***9***	***8***		***8***	***7***	***8***		***14***		***13***	
**H2O(w%) Eq.25b Plg hygrometer**	**0,3**	**0,4**	**0,3**	**0,5**		**0,6**	**0,6**	**0,5**	**0,5**	**0,8**		**0,8**	**0,7**	**0,7**		**0,2**		**0,5**	

**Table 3:** Selected microprobe analyses of plagioclase on Phase 2 samples (c: core. r: rim) (Calculated based on 32 O).

Plagioclase	GD-1							GD-2						GD-6			
	A2-1c	A2-1r	A3-2c	A4-2c	A4-2r	A5-1c	A7-1c	A3-1c	A4-1c	A4-1r	A5-1c	A10-1c	A10-1r	A6-1c	A6-2c	A7-1c	A7-1r
**SiO**_**2**_	56,15	53,27	53,85	56,40	54,45	58,48	56,17	53,56	58,51	57,10	53,48	54,41	59,07	52,32	54,15	54,51	53,14
**TiO**_**2**_	0,01	0,02	0,06	0,03	0,00	0,04	0,01	0,00	0,01	0,02	0,01	0,00	0,00	0,06	0,00	0,08	0,02
**Cr**_**2**_ **O**_**3**_	0,00	0,00	0,00	0,00	0,00	0,00	0,00	0,00	0,00	0,00	0,00	0,00	0,00	0,00	0,00	0,00	0,00
**Al**_**2**_ **O**_**3**_	25,99	27,00	27,51	26,56	27,21	24,20	24,81	28,57	23,19	25,47	28,98	28,09	25,00	29,33	28,02	27,20	28,82
**FeO**	0,47	0,52	0,48	0,45	0,53	0,37	0,41	0,34	0,32	0,32	0,36	0,35	0,24	0,41	0,31	0,39	0,50
**MnO**	0,00	0,00	0,00	0,00	0,00	0,00	0,00	0,00	0,00	0,00	0,00	0,00	0,00	0,00	0,00	0,00	0,00
**MgO**	0,08	0,02	0,09	0,11	0,06	0,01	0,10	0,01	0,05	0,01	0,08	0,03	0,03	0,07	0,06	0,08	0,06
**CaO**	11,98	12,93	11,60	10,85	11,47	10,10	10,53	11,05	9,79	10,67	11,60	11,15	8,16	11,39	11,84	11,07	11,93
**K**_**2**_ **O**	0,10	0,18	0,12	0,14	0,16	0,14	0,23	5,48	0,21	0,16	5,11	0,15	0,28	0,16	0,15	0,29	0,14
**Na**_**2**_ **O**	5,18	5,08	5,70	5,36	5,62	5,87	6,83	0,16	6,12	5,97	0,15	5,16	6,89	5,50	4,97	5,61	5,05
**Total**	99,97	99,02	99,39	99,90	99,50	99,19	99,08	99,17	98,20	99,73	99,77	99,33	99,66	99,24	99,50	99,24	99,66
**Si** ^**4+**^	10,18	9,73	9,75	10,21	9,86	10,66	10,15	9,72	10,76	10,32	9,67	9,89	10,62	9,47	9,84	9,89	9,84
**Ti** ^**4+**^	0,00	0,00	0,01	0,00	0,00	0,01	0,00	0,00	0,00	0,00	0,00	0,00	0,00	0,01	0,00	0,01	0,00
**Cr** ^**3+**^	0,00	0,00	0,00	0,00	0,00	0,00	0,00	0,00	0,05	0,00	0,00	0,00	0,00	0,00	0,00	0,00	0,00
**Al** ^**3+**^	5,55	5,81	5,87	5,67	5,81	5,20	5,28	6,11	5,02	5,43	6,18	6,02	5,30	6,26	6,00	5,82	6,00
**Fe** ^**2+**^	0,07	0,08	0,07	0,07	0,08	0,06	0,06	0,05	0,48	0,05	0,05	0,05	0,04	0,06	0,05	0,06	0,05
**Mn** ^**2+**^	0,00	0,00	0,00	0,00	0,00	0,00	0,00	0,00	0,00	0,00	0,00	0,00	0,00	0,00	0,00	0,00	0,00
**Mg** ^**2+**^	0,02	0,01	0,02	0,03	0,02	0,00	0,03	0,00	0,01	0,00	0,02	0,01	0,01	0,02	0,02	0,02	0,02
**Ca** ^**2+**^	2,33	2,53	2,25	2,10	2,23	2,08	2,04	2,15	1,93	2,07	2,25	2,17	1,57	2,21	2,31	2,15	2,31
**Na** ^**+**^	1,82	1,80	2,00	1,88	1,97	1,97	2,39	1,93	2,18	2,09	1,79	1,82	2,40	1,93	1,75	1,98	1,75
**K** ^**+**^	0,02	0,04	0,03	0,03	0,04	0,03	0,05	0,04	0,05	0,04	0,03	0,03	0,06	0,04	0,03	0,07	0,03
**Ab**	43,65	41,18	46,77	46,82	46,57	50,88	53,37	46,86	52,45	49,88	44,02	45,19	59,47	46,23	42,79	47,09	42,79
**An**	55,78	57,89	52,60	52,36	52,56	48,35	45,47	52,22	46,38	49,24	55,16	53,95	38,97	52,89	56,36	51,31	56,36
**Or**	0,57	0,93	0,63	0,82	0,87	0,77	1,16	0,92	1,17	0,88	0,83	0,86	1,57	0,88	0,85	1,60	0,85
**Kd (Ab-An) plg-liq =(0.28 ± 0.11)**	0,13	0,12	0,15	0,15	0,15	***0,18***	***0,20***	0,01	***0,23***	0,20	0,01	***0,17***	0,30	***0,17***	0,15	***0,18***	0,15
**T(°C) Eq.24a (± 36)**						***1131***	***1127***		***1131***			***1111***		***1138***		***1131***	
**P (kbar) Eq. 25a (± 2,5)**						***6***	***7***		***10***			***8***		***6***		***8***	
**H**_**2**_ **O(w%) Eq.25b Plg hygrometer**						**1,5**	**1,6**		**1,6**			**1,4**		**1,8**		**1,8**	

Olivine microcrysts (~100 µm) exhibit a narrower compositional range, with Fo contents of 68–81 (Table 4; Fig 12). Phase 1 contains euhedral olivines with normal zoning and Fo-rich cores. In Phase 2, orthopyroxene mesocrysts (~300 µm) have compositions corresponding to enstatite, with Wo₂–₁₂, En₅₆–₇₁, and Fs₂₆–₃₉ (Table 5; Figs 11b, 12f). Clinopyroxene microphenocrysts are augite, with Wo₃₄–₄₀, En₄₄–₄₈, and Fs₁₆–₁₈ (Table 5).

**Table 4:** Selected microprobe analyses of olivine (c: core. r: rim) in Phase 1 samples (Calculated based on 4 O).

Olivine	GD-3							GD-4						GD-5			
	A1-1c	A1-1r	A2-1c	A2-1r	A2-2c	A4-1c	A4-1r	A4-2c	A4-2r	A5-2c	A7-1c	A7-2r	A10-1c	A9-2c	A9-2r	A10-1c	A10-1r
**SiO**_**2**_	38,46	38,00	43,10	38,98	39,62	39,94	37,51	37,23	38,49	39,21	39,29	39,72	36,55	39,39	43,01	36,76	37,42
**TiO**_**2**_	0,03	0,03	0,00	0,00	0,05	0,06	0,05	0,00	0,00	0,09	0,00	0,08	0,00	0,01	0,04	0,05	0,03
**Al**_**2**_ **O**_**3**_	0,04	0,05	0,01	0,00	0,01	0,08	0,05	0,07	0,04	0,08	0,03	0,09	0,05	0,05	0,04	0,11	0,29
**Cr**_**2**_ **O**_**3**_	0,01	0,06	0,02	0,00	0,01	0,00	0,00	0,03	0,01	0,00	0,05	0,04	0,00	0,05	0,00	0,00	0,00
**FeO**	22,44	21,21	16,51	21,69	19,21	20,56	21,02	20,30	20,76	27,19	20,96	20,66	25,61	22,56	19,53	18,73	20,43
**MgO**	38,24	40,37	39,82	39,07	40,41	39,46	41,79	41,23	40,03	32,82	38,51	38,19	36,26	38,20	36,44	43,28	41,92
**CaO**	0,27	0,22	0,16	0,23	0,18	0,24	0,23	0,17	0,25	0,30	0,25	0,24	0,28	0,25	0,23	0,14	0,26
**Na**_**2**_ **O**	0,00	0,00	0,03	0,16	0,00	0,00	0,00	0,00	0,09	0,00	0,03	0,08	0,05	0,02	0,05	0,06	0,00
**K**_**2**_ **O**	0,01	0,01	0,03	0,00	0,00	0,00	0,00	0,00	0,00	0,00	0,00	0,01	0,00	0,01	0,00	0,02	0,01
**Total**	99,50	99,94	99,67	100,12	99,48	100,33	100,64	99,04	99,67	99,68	99,12	99,10	99,88	100,54	99,33	99,13	100,36
**Si**	1,00	0,98	1,08	1,01	1,02	1,02	0,97	0,97	1,00	1,04	1,02	1,03	0,98	1,02	1,10	0,95	0,96
**Fe** ^**2+**^	0,49	0,46	0,35	0,47	0,41	0,44	0,45	0,44	0,45	0,60	0,46	0,45	0,57	0,49	0,42	0,41	0,44
**Mn**	0,00	0,00	0,00	0,00	0,00	0,00	0,00	0,00	0,00	0,00	0,00	0,00	0,00	0,00	0,00	0,00	0,00
**Mg**	1,49	1,56	1,49	1,51	1,55	1,50	1,60	1,60	1,55	1,30	1,49	1,48	1,45	1,47	1,38	1,67	1,61
**Ca**	0,01	0,01	0,00	0,01	0,01	0,01	0,01	0,00	0,01	0,01	0,01	0,01	0,01	0,01	0,01	0,00	0,01
**Fo**	75,23	77,24	81,14	76,26	78,95	77,39	77,99	78,36	77,46	68,28	76,61	76,72	71,62	75,12	76,89	80,47	78,54
**Fa**	24,77	22,76	18,86	23,74	21,05	22,61	22,01	21,64	22,54	31,72	23,39	23,28	28,38	24,88	23,11	19,53	21,46
**Tp**	0,00	0,00	0,00	0,00	0,00	0,00	0,00	0,00	0,00	0,00	0,00	0,00	0,00	0,00	0,00	0,00	0,00
**Kd (Fe-Mg)** ^**ol-melt**^ **= (0,27-0,33)**	0,36	0,32	0,26	0,34	***0,29***	***0,29***	0,31	0,17	0,18	***0,32***	0,19	0,20	***0,27***	0,21	0,19	0,15	0,17
***T (°C) [46] Eq. 4 (± 29)***					***1229***	***1235***				***1167***			***1153***				
**T (°C) *[47]* (± 55)**					***1253***	***1253***				***1172***			***1172***				
Mg# (liq)	0,52	0,52	0,52	0,52	0,52	0,52	0,52	0,41	0,41	0,41	0,41	0,41	0,41	0,39	0,39	0,39	0,39
Mg# (fo)	0,75	0,77	0,81	0,76	0,79	0,77	0,78	0,78	0,77	0,68	0,77	0,77	0,72	0,75	0,77	0,80	0,79

**Table 5:** Selected microprobe analyses of orthopyroxene and clinopyroxene (c: core. r: rim) in Phase 2 samples (Calculated based on 6 O).

	Ortopyroxene														Clinopyroxene		
	GD-1									GD-2		GD-6			GD-2		
	A2-1c	A2-2c	A2-2r	A4-1c	A4-1r	A7-1c	A7-1r	A9-1c	A10-1r	A3-1c	A3-1r	A5-1c	A6-2c	A9-1c	A2-1c	A2-1r	A2-2c
**SiO**_**2**_	54,89	54,69	52,41	53,30	54,88	51,17	55,39	54,68	53,75	55,77	53,50	53,42	53,79	53,07	53,42	52,10	55,88
**TiO**_**2**_	0,19	0,34	0,17	0,26	0,23	0,29	0,25	0,17	0,23	0,28	0,19	0,07	0,28	1,10	0,31	0,48	0,29
**Cr**_**2**_ **O**_**3**_	0,00	0,00	0,00	0,01	0,00	0,00	0,03	0,00	0,02	0,00	0,00	0,00	0,00	0,00	0,04	0,04	0,22
**Al2O3**	1,95	1,88	2,31	1,82	2,77	1,99	3,48	0,88	1,71	6,37	2,12	0,64	1,88	3,45	1,59	1,47	1,71
**FeO**	16,85	16,75	18,44	17,21	16,18	22,93	19,11	18,12	18,61	16,12	22,54	18,25	22,16	22,50	9,78	10,45	10,54
**MnO**	0,00	0,00	0,00	0,00	0,00	0,00	0,00	0,00	0,00	0,00	0,00	0,00	0,00	0,00	0,00	0,00	0,00
**MgO**	23,67	24,10	24,47	26,22	23,82	22,36	18,18	23,09	23,43	15,51	18,41	26,58	18,98	16,80	15,38	16,13	15,62
**CaO**	1,95	1,91	1,86	1,42	1,26	1,53	0,91	2,77	1,69	4,59	2,10	1,43	1,73	2,65	19,40	18,41	15,18
**K**_**2**_ **O**	0,03	0,02	0,00	0,01	0,02	0,05	0,34	0,02	0,01	0,10	0,13	0,00	0,10	0,55	0,00	0,01	0,13
**Na**_**2**_ **O**	0,20	0,00	0,00	0,05	0,17	0,05	0,78	0,03	0,11	0,86	0,19	0,06	0,18	0,80	0,18	0,23	0,39
**Total**	99,72	99,68	99,66	100,30	99,34	100,37	98,46	99,75	99,55	99,59	99,17	100,47	99,13	100,90	100,10	99,31	99,96
**Si** ^**4+**^	2,01	2,00	1,92	1,92	2,01	1,89	2,10	2,02	1,98	2,09	2,03	1,93	2,04	1,99	1,98	1,94	2,08
**Al** ^**3+**^	0,00	0,00	0,08	0,08	0,00	0,09	0,00	0,00	0,02	0,00	0,00	0,03	0,00	0,01	0,02	0,06	0,00
**Sum T (IV)**	2,01	2,00	2,00	2,00	2,01	1,98	2,10	2,02	2,00	2,09	2,03	1,96	2,04	2,00	2,00	2,00	2,08
**Ti** ^**4+**^	0,01	0,01	0,00	0,01	0,01	0,01	0,01	0,00	0,01	0,01	0,01	0,00	0,01	0,03	0,01	0,01	0,01
**Al** ^**3+**^	0,08	0,08	0,02	0,00	0,12	0,00	0,15	0,04	0,06	0,28	0,09	0,00	0,08	0,14	0,05	0,01	0,07
**Cr**	0,00	0,00	0,00	0,00	0,00	0,00	0,00	0,00	0,00	0,00	0,00	0,00	0,00	0,00	0,00	0,00	0,01
**Fe3** ^**+**^	0,00	0,00	0,05	0,06	0,00	0,11	0,00	0,00	0,00	0,00	0,00	0,11	0,00	0,00	0,00	0,04	0,00
**Fe** ^**2+**^	0,26	0,25	0,25	0,19	0,23	0,33	0,35	0,31	0,31	0,26	0,43	0,17	0,44	0,47	0,25	0,22	0,23
**Mg**	0,64	0,65	0,67	0,70	0,62	0,60	0,46	0,65	0,65	0,39	0,51	0,71	0,52	0,46	0,69	0,71	0,60
**Sum M1 (VI)**	0,99	0,99	0,99	0,96	0,98	1,05	0,97	1,00	1,02	0,94	1,04	0,99	1,05	1,09	1,00	0,99	0,92
**Fe** ^**2+**^	0,26	0,26	0,26	0,27	0,27	0,27	0,26	0,25	0,26	0,24	0,29	0,27	0,26	0,24	0,06	0,06	0,10
**Mn**	0,00	0,00	0,00	0,00	0,00	0,00	0,00	0,00	0,00	0,00	0,00	0,00	0,00	0,00	0,00	0,00	0,00
**Mg**	0,65	0,67	0,67	0,71	0,68	0,63	0,57	0,62	0,64	0,48	0,53	0,72	0,55	0,48	0,16	0,19	0,27
**Ca**	0,08	0,07	0,07	0,05	0,05	0,06	0,04	0,11	0,07	0,18	0,09	0,06	0,07	0,11	0,77	0,74	0,60
**K**	0,00	0,00	0,00	0,00	0,00	0,00	0,02	0,00	0,00	0,00	0,01	0,00	0,01	0,06	0,00	0,00	0,01
**Na**	0,01	0,00	0,00	0,00	0,01	0,00	0,06	0,00	0,01	0,06	0,01	0,00	0,00	0,03	0,01	0,02	0,03
**Sum M2 (VI)**	1,00	1,01	1,01	1,04	1,01	0,96	0,94	0,98	0,98	0,97	0,93	1,05	0,90	0,91	1,00	1,01	1,00
**Total**	4,00	4,00	4,00	4,00	4,00	4,00	4,00	4,00	4,00	4,00	4,00	4,00	4,00	4,00	4,00	4,00	4,00
**Wo**	4,06	3,94	3,69	2,76	2,69	3,03	2,22	5,64	3,46	11,85	4,63	2,72	3,81	6,09	40,06	37,56	33,64
**En**	68,56	69,11	67,69	71,07	70,46	61,55	61,51	65,51	66,79	55,68	56,53	70,22	58,12	53,62	44,18	45,79	48,14
**Fs**	27,38	26,96	28,61	26,17	26,86	35,41	36,27	28,85	29,75	32,47	38,83	27,05	38,07	40,29	15,76	16,64	18,23
**Kd(Fe-Mg)** ^**opx−liq**^ **= (0,29 ± 0,06)**	0,20	0,20	0,21	0,19	0,19	0,24	0,30	0,22	0,23	***0,24***	0,28	0,19	0,23	***0,26***			
**T(°C)Eq.28a (± 39)**										***1110***				***1011***			
**P (kbar) Eq. 29b (± 2,1)**										***5***				***4***			
**Kd(Fe-Mg)**^**cpx-liq**^ **(0.27 ± 0.03)**															0,23	0,24	***0,25***
**T(°C) Eq.32d (± 42)**																	***1147***
***P (kbar) Eq. 32a*&*b (± 1,5)***																	***6***
Mg# (liq)	0,34	0,34	0,34	0,34	0,34	0,34	0,34	0,34	0,34	0,29	0,29	0,26	0,26	0,26	0,29	0,29	0,29
Mg# (opx)	0,71	0,72	0,70	0,73	0,72	0,63	0,63	0,69	0,69	0,63	0,59	0,72	0,60	0,57			
Mg# (cpx)															0,74	0,73	0,73

## 7. Thermobarometric estimations

**Fig 13:**
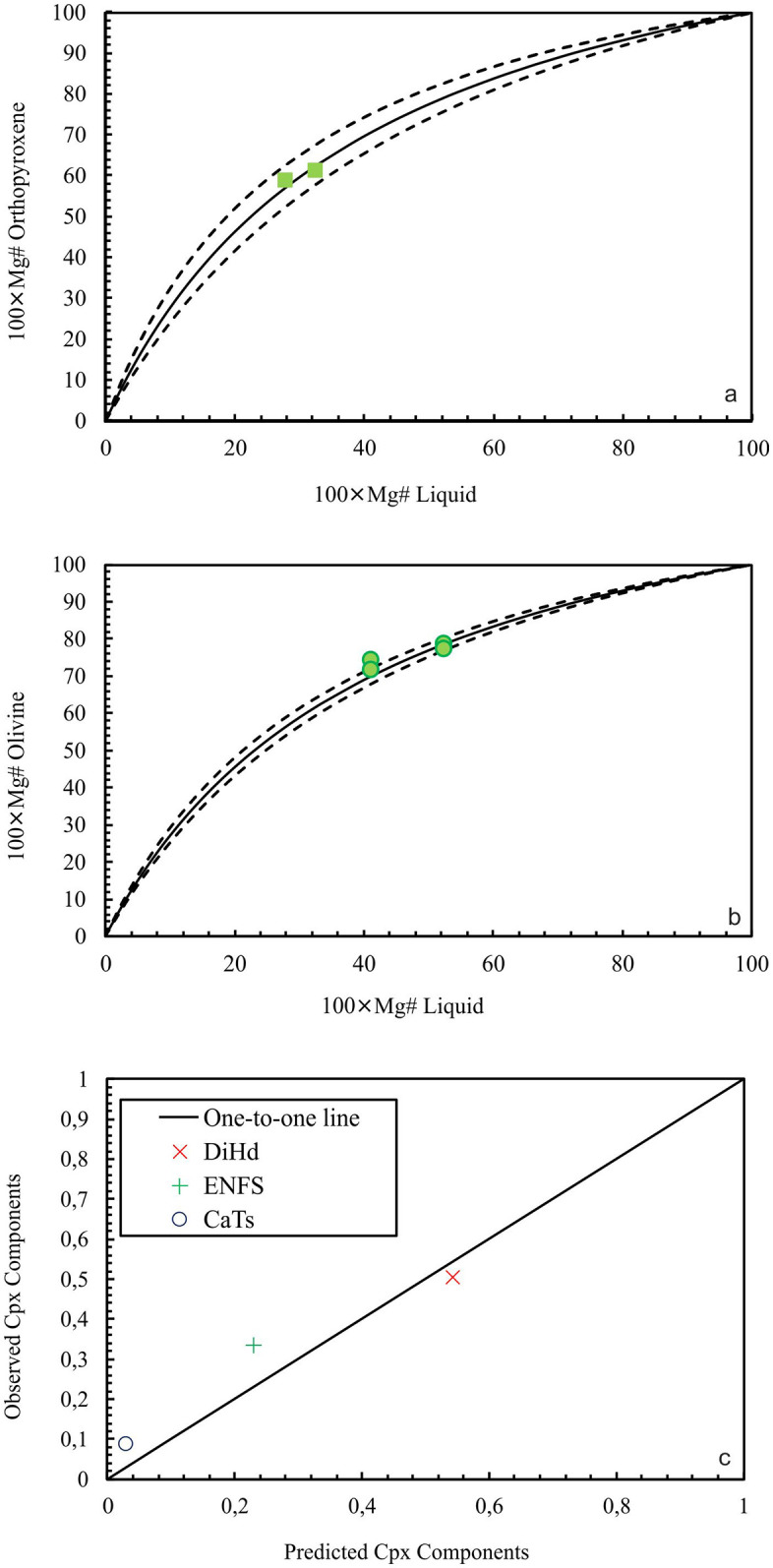
The Rhodes equilibrium diagrams (a) opx–melt, (b) olivine–melt, (c) equilibrium tests for clinopyroxene based on observed (mineral) vs. predicted (melt) (c).

The P–T conditions of Phases 1 and 2 were determined using plagioclase (Table 2 and Table 3), which is present in both phases along with olivine (Table 4) in Phase 1 and orthopyroxene and clinopyroxene (Table 5) in Phase 2. Equilibrium plagioclase macrocrysts were analyzed to estimate the P-T conditions and depths of the magma storage regions for both phases (Table 6). [45] recommends using plagioclase barometer estimates together with independent pressure values derived from other mineral–melt equilibrium models. For this reason, two-pyroxene thermometer was tested; however, none of the pairs were in equilibrium. Based on this principle, P–T conditions calculated using equilibrium olivine crystals from Phase 1, which were used exclusively for temperature (T) estimations, as well as the commonly observed orthopyroxenes and the less abundant clinopyroxenes from Phase 2. The Rhodes equilibrium diagrams forming the basis of the P–T calculations for opx–melt (Fig 13a) and olivine–melt (Fig 13b), along with the equilibrium tests for clinopyroxene based on observed (mineral) vs. predicted (melt) compositions of diopside–hedenbergite, enstatite–ferrosilite, and Ca–Tschermakite components, are presented in Fig 13c.

**Table 6:** Estimated temperature (°C), pressure (kbar) and depth (km) values for Phase 1 and Phase 2 flows calculated after [45,46] and [47].

Phase 1				
Plagioclase				
*P (kbar)*	*T (°C)*	*depth (km)*		
[45] *Eq. 25a (± 2.5)*	[45] *Eq.24a (± 36)*	*(d=2.50 kg/cm*^*3*^)	*(d=2.90 kg/cm*^*3*^)	*Average(d=2.70 kg/cm*^*3*^)
10	1186	41	35	38
**Olivine**				
***T (°C)***	***T (°C)***			
[46] *Eq. 4 (± 29)*	[47]*(± 55)*			
1196	1213	–		
**Phase 2**				
**Plagioclase**				
***P (kbar)***	***T (°C)***	***depth (km)***		
[45] *Eq. 25a (± 2.5)*	[45]*Eq.24a (± 36)*	*(d=2.50 kg/cm*^*3*^)	*(d=2.90 kg/cm*^*3*^)	*Average(d=2.70 kg/cm*^*3*^)
8	1128	33	28	30
**Clinopyroxene**				
***P (kbar)***	***T (°C)***	***depth (km)***		
[45] *Eq. 32a*&*b (± 1.5)*	[45] *Eq.32d (± 42)*	*(d=2.50 kg/cm*^*3*^)	*(d=2.90 kg/cm*^*3*^)	*Average(d=2.70 kg/cm*^*3*^)
6	1147	24	21	23
**Orthopyroxene**				
***P (kbar)***	***T (°C)***	***depth (km)***		
[45] *Eq. 29b (± 2.1)*	[45] *Eq.28a (± 39)*	*(d=2.50 kg/cm*^*3*^)	*(d=2.90 kg/cm*^*3*^)	*Average(d=2.70 kg/cm*^*3*^)
5	1061	20	18	19

In Phase 1, temperature was calculated as 1186 ± 36 °C from equilibrium core of the plagioclase crystals based on the Rhodes diagram (Kd _(Ab–An)_ = 0.28 ± 0.11) using [45] Equation 24a, which also yielded a pressure of 10 kbar. Equilibrium olivine crystals (Kd _(Fe–Mg)_ = 0.264–0.292) yielded a temperature of 1196 ± 29 °C using [46] Equation 4, consistent with the plagioclase-based estimate. An additional calculation using the [47] correction produced a temperature of 1213 ± 55 °C. For Phase 2, the temperature calculated from equilibrium plagioclase crystals (Kd _(Ab–An)_ = 0.28 ± 0.11), based on the Rhodes diagram and using [45] Equation 24a, is 1128 ± 36 °C, and the pressure estimated from the same equation is 8 kbar. Equilibrium orthopyroxene crystals (Kd _(Fe–Mg)_ = 0.253 and 0.342) yield a temperature of 1061 °C using Equation 28 of [45], and a pressure of 5 kbar using Equation 29b. In addition, clinopyroxene, which is less commonly observed in Phase 2, yields a temperature of 1147 °C from the core of an equilibrium grain (Kd _(Fe–Mg)_ = 0.25) using Equation 32d, while Equations 32a and 32b give a pressure of 6 kbar. The reported uncertainties for the thermobarometric represent the standard error of estimate (SEE) of the calibration.

Temperature estimates derived from Phase 1, as well as the pressure calculated from plagioclase, are higher than those obtained for Phase 2. The lithostatic pressure equation (P = ρ·g·h) was used to calculate depth, based on upper crustal densities of 2.50 and 2.90 g/cm³, which are typical for Eastern Anatolia, [48–56], and a gravitational acceleration of 9.81 m/s². In addition, I used the average of the two density values (2.70 g/cm³) to calculate the depths and constructed my model accordingly (Table 6). Based on the 2.70 g/cm^3^ density, the depth estimated only from plagioclase for Phase 1 is ~ 38 km. In Phase 2, the temperatures estimated from plagioclase, clinopyroxene, and orthopyroxene are relatively consistent when considering analytical uncertainties. The corresponding pressure estimates from these minerals using the same density and a gravitational acceleration, the calculated depths are 30 km, 23 km, and 19 km, respectively. The distinction between these magmatic systems suggest that differentiation played a significant role in their evolution (Fig 7).

## 8. Discussion

### 8.1. Geochemical constraints on the magmatic evolution

Comparing the samples from this study with Mount Ağrı volcanic rocks reported in the literature may provide insights into the magmatic evolution of the region. According to the TAS diagram (Fig 6), the samples cover a wide compositional range from mildly alkaline- transitional and sub-alkaline basaltic flows to sub-alkaline intermediate to acidic (andesite-dacite) flows in [40], andesite to dacite in [39], and transitional alkali basalt to basaltic andesite in [38]. In light of literature, Phase 2 exhibits distinct geochemical affinities with several previously studied volcanic units, emphasizing its close compositional relationship to the broader regional magmatic system. Harker diagrams (Fig 7), show that Phase 1 and Phase 2 follow similar trends to the comparison data. Phase 1, with high FeO*, TiO₂, CaO, and Al₂O₃ contents at low SiO₂, represents less evolved basaltic–andesitic compositions, whereas Phase 2, with lower FeO*, TiO₂, and CaO contents at high SiO₂, reflects more evolved andesitic–dacitic compositions that show evidence of advanced fractional crystallization. These differences suggest that both phases were derived from a similar source but show evidence of distinct magmatic evolution processes.

Fig 8 presents primitive mantle–normalized trace element patterns comparing Phase 1 and Phase 2 samples from this study, compared with literature data. Both phases are characterized by pronounced enrichment in LILEs (Cs, Rb, Ba, Th, U, K), Nb–Ta negative anomalies, and moderately fractionated HREEs, reflecting subduction-enriched mantle sources. Phase 2 differs from Phase 1 by having higher LILE and LREE abundances and more distinct Nb–Ta trends. Phase 1 generally displays lower trace element abundances and a flatter pattern, which may be related to a higher degree of partial melting or a more depleted source. In the literature, the Cumaçay volcanics of [40] show patterns closer to Phase 2, [39] exhibit similarities to both phases, and [38] more closely resemble Phase 1. Almost all comparative samples and the samples from this study, are characterized by Nb–Ta depletions and low HREE contents, features that indicate a mantle source modified by subduction processes. REE patterns (Fig 9) exhibit LREE enrichment and relative HREE depletion, indicating a subduction-metasomatized, enriched mantle source. The parallel trends of the samples suggest that partial melting played a more dominant role than magmatic fractionation. The absence of a marked negative Eu anomaly further supports the interpretation that neither intense plagioclase fractionation nor significant crustal contamination occurred. These trends indicate that the magmas in the study area were generated at varying depths and degrees of partial melting within the same subduction-modified mantle source.

### 8.2. Implications of thermobarometric results

Magma cooling and crystallization paths are controlled by several factors, including P-T, composition, volatile content, and magma chamber geometry [57]. The resolution of seismic methods is insufficient to detect the spatial geometry of small-volume magma chambers, while petrological investigations rely on mineral phases and melt inclusions as the memory of P, T and compositional parameters. However, these records may be modified during the various stages of magma ascent and evolution [58]. During magma storage, evolution and ascent, phenocrysts in equilibrium with the melt can be used to estimate the magma’s temperature (T) and pressure (P) [59]. In this study, mineral–melt equilibrium conditions are evaluated on the basis of [45]. Thermobarometric results indicate that Phase 1 magmas crystallized at higher temperatures (~1185–1215 °C) compared to Phase 2 (~1060–1150 °C). In Phase 2, P–T estimations can be derived from multiple mineral phases, which provides a more robust basis for interpretation. These differences highlight the contrasting crystallization conditions of the two phases, with Phase 1 representing hotter and more mafic magmas, while Phase 2 records cooler and more evolved compositions. For Phase 1 (LOI = 0.10–0.30 wt.%) and Phase 2 (LOI = 0.10–0.70 wt.%), mineral–melt equilibrium and thermobarometric estimations were carried out assuming a hydrous melt composition. The estimated water content (H₂O wt.%) calculated using the plagioclase hygrometer of [45] (Eq. 25b), based on the silica content of the melt and the chemical parameters of plagioclase and the liquid. The results indicate H₂O contents of 0.3–0.8 wt.% for Phase 1 and 1.5–1.8 wt.% for Phase 2 (Table 2; Table 3). The higher water content of Phase 2 is consistent with its glassy texture and abundant fine-grained microlites, which suggest enhanced undercooling. Both the initial temperature and water content play a key role in controlling undercooling [60]. As noted by [61], an increase in water content promotes rapid microlite crystallization due to enhanced cation diffusivity in silicate melts. Accordingly, Phase 2 contains a greater abundance of fine-grained microlites and comparatively fewer phenocrysts and macrocrysts than Phase 1, reflecting its rapid cooling history.

### 8.3. Magma differentiation and storage zones

The differentiation process from basaltic Phase 1 to andesitic Phase 2 represents a classical magmatic evolution pattern. Phase 1 samples represent a more primitive, mafic while Phase 2 samples reflect more evolved, intermediate magma. Basaltic magmas form through partial melting of the mantle. The range from basic to acidic magmas is common in volcanic arcs, especially in subduction zones where the interaction of the mantle wedge with water and other volatiles triggers the formation of basaltic magmas undergoing differentiation. Previous studies and limited trace element data from this study on Mount Ağrı have supported a significant subduction component in the lavas [1,39,62–64]. Additionally, geochemical and petrological studies from Eastern Anatolia suggest that, from the Miocene to the Quaternary, magmatism was increasingly influenced by melts derived from the lithospheric mantle, rather than from the asthenospheric mantle [39,40,65–67]. Additionally, the coexistence of transitional and sub-alkaline lavas within the Mount Ağrı system can be attributed to subsequent magmatic differentiation, with contributions from both primitive mantle-derived melts and subduction-modified sources. Moreover, [68] reported that Eastern Anatolian volcanism displays a north–south contrast: southern volcanoes (e.g., Süphan) are transitional, while northern centers (e.g., the Erzurum–Kars Plateau and Mount Ağrı) are mostly calc-alkaline with a stronger subduction influence. This geochemical signature diminishes to the south, where lavas become more alkaline and within-plate in character. AFC modeling suggests enhanced magma–crust interaction in the south, and radiometric data indicate that volcanism initiated earlier in the north ~11 Ma [17] before migrating southward.

**Fig 14:**
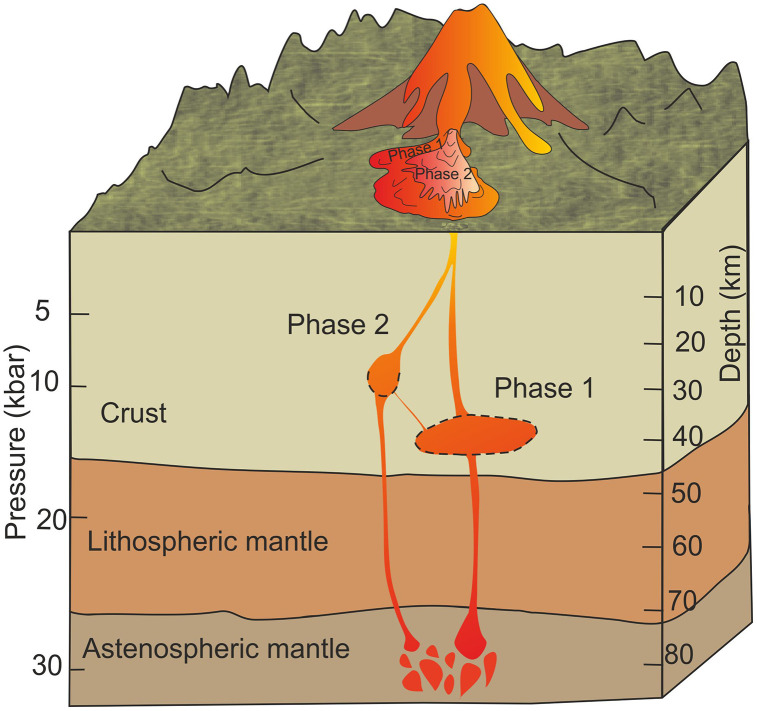
Schematic diagram of Phase 1 and Phase 2 magma plumbing system beneath Mount Ağrı. Crustal thickness and lithospheric mantle thickness for East Anatolia from [72]. The deeper magma reservoir of Phase 1 is illustrated with dashed lines, with its upper and lower boundaries constrained by plagioclase–melt P–T estimates, which [45] noted should be applied with caution. The shallower magma storage zone of Phase 2 is constrained by P–T estimates from plg–melt, cpx–melt, and opx–melt thermometers, and only the dashed line from the plg–melt estimate is used to indicate its lower boundary.

Recent studies on volcanoes in Eastern Anatolia suggest the presence of not only shallow magma chambers but also deeper magma reservoirs. Based on P-T estimates obtained in this study, the depths inferred from crystals in apparent equilibrium with the melt (plg, ol, opx and cpx) are estimated to be approximately ~40 km for Phase 1 and ~24 km for Phase 2 (Fig 14). The Phase 1 magma reservoir is shown with dashed lines, with boundaries constrained by plagioclase–melt P–T estimates that should be interpreted with caution [45]. Similarly, Phase 2 is constrained by multiple thermometers (plg–melt, cpx–melt, and opx–melt), although only the dashed line from the plg–melt estimate is used to represent its lower boundary. The presence of both deep magma reservoirs and shallow magma chambers is consistent with previous geophysical studies [69–71]. The 2.5-dimensional (2.5-D) gravity models of Eastern Anatolia [72], integrated with receiver function and seismic tomography data [56,73,74], indicate that the lithospheric mantle beneath the region ranges between 44 and 73 km in thickness. Petrographic observations and volcanological context suggest that Phase 1 and Phase 2 represent magmas that evolved under markedly different storage conditions. The progressive segregation of early-crystallized mafic minerals (olivine and pyroxene) from the melt within the plumbing system facilitated the formation of Phase 1, a process that led to silica enrichment and the generation of more differentiated andesitic compositions such as Phase 2. In particular, Phase 2 magmas were likely emplaced after recharge of the deeper reservoir, with subsequent transfer to intermediate and shallow storage zones [75]. Shorter residence times and more rapid ascent limited crystal growth, resulting in lower crystal contents and in some cases glassy groundmasses. Additionally, the crustal thickness of the Eastern Anatolia increases from 33 km in the west to 46 km in the east [72], while other studies have reported values ranging from 38 to 45 km [56,73,76,77]. This allows magma ascend through the lithosphere, rapidly arrives in the crust leading to episodic volcanic activity. Volcanologically, this contrast aligns with the distribution of differentiated lavas in the central part of Mount Ağrı and more basaltic compositions along the flanks [12], reflecting the interplay between reservoir geometry, rheological heterogeneities, and magma migration pathways. Furthermore, the results of this study indicate the presence of both a deep magma reservoir and a shallow magma chamber southeast of Mount Ağrı, which were active during the Quaternary period (~60.0 to ~20.0 ka).

### 8.4. Comparison with other volcanoes in Eastern Anatolia

Stratovolcanoes in Turkey are commonly fed by shallow crustal magma chambers [78], and the depths of shallow magma chambers are commonly located within a few kilometers of the ground surface [79]. Other stratovolcanoes in Eastern Anatolia, such as Nemrut have a shallow magma chamber ~ 6 km [80,81]. Additionally, seismic tomography in the Karlıova region reveals that Tunadağ and Varto volcanoes, which predominantly exhibit an intermediate composition, are associated with shallower magma chambers at depths of 2–4 km and 2–5 km, respectively. In contrast, Özenç volcano, characterized by a basaltic composition, is linked to a deeper magma chamber at a depth of ~22–27 km [82]. Another Plio-Quaternary volcanic eruption center, Cumaçay located 50 km northwest of Ağrı, mineral–melt equilibrium calculations derived from olivine, orthopyroxene, clinopyroxene, and plagioclase indicate crystallization occurred at deep ~14–28 km and shallow ~5–15 km magma chambers [40]. These studies, along with the results of this research, indicate that the volcanic centers in Eastern Anatolia have both shallow magma chambers and deep magma reservoirs, feeding different volcanic eruptions over time. This configuration can be defined as a vertically-stacked transcrustal magmatic system (Fig 14), a term originally defined and first introduced by [57].

## 9. Conclusion

This study investigates the pressure-temperature (P-T) conditions and depths of two distinct magma storage zones beneath southeast Mount Ağrı. Through mineralogical, geochemical analyses with ^40^Ar-^39^Ar whole-rock dating, the volcanic history and characteristics of two lava flows were examined. In addition, fieldwork highlighted that these lava flows exhibit significant macroscopic differences. Phase 1, a gray trachybasalt (49.2–51.2% SiO₂) with a scoriaceous texture, 57.70 ± 21.44 ka and contains plagioclase + olivine ± oxide minerals. Phase 2, a black andesite (62.42–63.4% SiO₂) with a smooth, glass-like surface, erupted at 19.09 ± 5.59 ka and consists of plagioclase + orthopyroxene ± clinopyroxene ± oxide minerals. The new geochronological and geochemical results clarified the compositional features of both flows and allowed their accurate placement within the stratigraphy. Both phases display Nb–Ta depletions and low HREE abundances, indicating a mantle source influenced by subduction-related processes. P–T estimates indicate that Phase 1 began crystallization at higher temperatures (up to ~1200 °C), consistent with the presence of abundant phenocrysts and macrocrysts that likely grew under relatively low undercooling conditions. In contrast, Phase 2 crystallized at lower temperatures (~1150 °C) and developed a finer-grained, more glassy texture, reflecting higher degree of undercooling. The presence of mainly normal and minor reverse zoning in plagioclase crystals suggests the absence of significant P–T variations.

The geochemical and mineralogical-petrological data suggest that Mount Ağrı has a hybrid magmatic system where deep-seated basaltic magmas supply shallow magma storage zones [12],[83], leading to progressive differentiation. The lack of amphibole in the Phase 1 and 2 samples is compatible with previous geochemical interpretations that suggest amphibole crystallization occurs at greater depths [62,84]. Accordingly, Dy/Yb decreases from 1.88 in Phase 1 to 1.71 in Phase 2, a trend that can be attributed to amphibole fractionation. Amphibole is expected to be a liquidus phase in basaltic to basaltic – andesite magmas under near water-saturated conditions (> 10 wt.% H_2_0 at 15 kbar) between depths of around 25–80 km (8–25 kbar). If the same magma ascends to shallower crustal levels, the liquidus phases become plagioclase and two pyroxenes, and any entrained amphibole crystals are resorbed by the melt [17]. As a whole, these findings confirm the existence of a shallow magma chamber (~24 km) and a deeper magma reservoir (~40 km) that were active at different times during the Quaternary, producing both mafic and intermediate volcanic products at varying crustal levels.
